# QuickStats

**Published:** 2014-06-20

**Authors:** FROM THE NATIONAL CENTER FOR HEALTH STATISTICS

**Figure:**
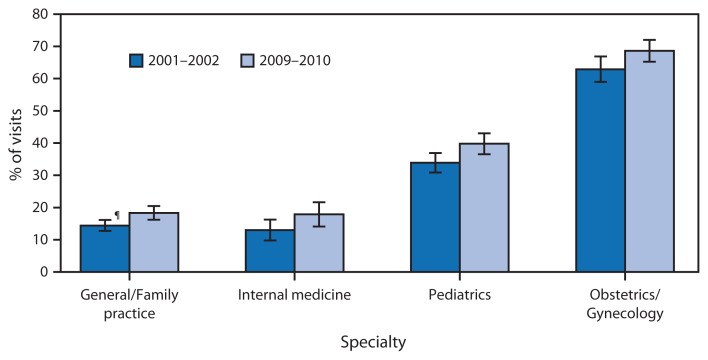
Percentage of Physician Office Visits* for Which Preventive Care^†^ Was the Major Reason for Visit, by Selected Specialties^§^ — National Ambulatory Medical Care Survey, United States, 2001–2002 and 2009–2010 * Percentages are 2-year annual averages. Visits to community health centers were excluded from this analysis. ^†^ The National Ambulatory Medical Care Survey defines physician office visits for which preventive care was the major reason for visit as “General medical examinations and routine periodic examinations. Includes prenatal and postnatal care, annual physicals, well-child examinations, screening, and insurance examinations.” Immunizations might or might not be administered during the visit. ^§^ Subspecialties of physician specialty categories listed were excluded. ^¶^ 95% confidence interval.

From 2001–2002 to 2009–2010, the percentage of physician office visits for which preventive care was the major reason for visit increased for the specialties of general/family practice, internal medicine, pediatrics, and obstetrics and gynecology. During 2009–2010, approximately two thirds of visits to obstetricians and gynecologists were for preventive care, including prenatal and postnatal care, and more than one third of visits to pediatricians were for preventive care.

**Source:** National Ambulatory Medical Care Survey 2001–2002 and 2009–2010. Available at http://www.cdc.gov/nchs/ahcd.htm.

**Reported by:** Michael Albert, MD, wmy1@cdc.gov, 301-458-4223; Linda F. McCaig, MPH.

